# Antiproliferative and cytotoxic effects of grape pomace and grape seed extracts on colorectal cancer cell lines

**DOI:** 10.1002/fsn3.1150

**Published:** 2019-08-02

**Authors:** José M. Pérez‐Ortiz, Luis F. Alguacil, Elisabet Salas, Isidro Hermosín‐Gutiérrez, Sergio Gómez‐Alonso, Carmen González‐Martín

**Affiliations:** ^1^ Unidad de Investigación Traslacional Hospital General Universitario de Ciudad Real Ciudad Real Spain; ^2^ Instituto Regional de Investigación Científica Aplicada (IRICA) Universidad de Castilla‐La Mancha Ciudad Real Spain; ^3^ Department of food science and technology Universidad de Castilla‐La Mancha Ciudad Real Spain; ^4^ Present address: Facultad de Farmacia Universidad CEU San Pablo Madrid Spain; ^5^ Present address: European Commission, Research Executive Agency Brussels Belgium

**Keywords:** antitumoral, Caco‐2, grape extracts, HT‐29

## Abstract

Grape pomace is the source of bioactive compounds (anthocyanins, flavonols, flavan‐3‐ols, and stilbenes) which exhibit antiproliferative actions on cell cultures. We have investigated the antitumoral effects of grape pomace and grape seed extracts on colon cancer cells (Caco‐2, HT‐29) and fibroblasts. Crude extracts prepared from white and red pomace, and grape seeds, reduced the viability and proliferation of Caco‐2. HT‐29 cells were resistant to these actions. Purified extracts were then prepared from the same sources and compared with the LDH test; again, all three extracts were active and purified extract from grape seed was the most potent and specific on Caco‐2 cells. HT‐29 cells were more sensitive to these purified extracts. The biological activity resided almost exclusively in the flavonol and flavan‐3‐ols subfractions, rather than the anthocyanin subfraction. Preliminary results on the mechanisms involved in these effects revealed downregulation of Myc gene expression in HT‐29 and upregulation of Ptg2 in Caco‐2 cells.

## 1 INTRODUCTION

Great effort has been directed to isolate and characterize potential chemopreventive agents present in vegetables and fruits, since the latter have been hypothesized to be major dietary contributors to cancer prevention (Glade, 1999). Many of these phytochemicals have shown promising anticancer efficacy in cell cultures and animal models, including bioflavonoids such as catechins, proanthocyanidins, phytoestrogens, and other phenolic compounds (Amico et al., 2009; Faria, Calhau, de Freitas, & Mateus, 2006; Fresco, Borges, Marques, & Diniz, 2010; Khan, Adhami, & Mukhtar, 2009; Lamoral‐Theys et al., 2010; Liu, Jiang, & Xie, 2010; Sun, Yuan, Koh, & Yu, 2006).

The most common industrial methods to obtain phenolic extracts from grape pomace are based on conventional solid–liquid extraction with ethanol–water solutions or sulfite‐containing water (Sefcal process; Gonzalez‐Paramas, Esteban‐Ruano, Santos‐Buelga, de Pascual‐Teresa, & Rivas‐Gonzalo, 2004). Different alternative procedures have been tested in the last decades: the use of hydroalcoholic mixtures in different proportions and temperatures, including extraction assisted by different systems such as autoclaves or soxhlet extraction (Campos, Leimann, Pedrosa, & Ferreira, 2008; Cruz, Conde, Domínguez, & Parajó, 2007; Cruz, Domínguez, & Parajó, 2004); enzymatic treatments that produce the degradation of cell wall polysaccharides (Maier, Göppert, Kammerer, Schieber, & Carle, 2008; Meyer, Jepsen, & Sørensen, 1998); new technologies such as supercritical fluid extraction with CO_2_ or modified CO_2_ (Campos et al., 2008; Pinelo et al., 2007); superheated liquid extraction (Luque‐Rodríguez, Luque de Castro, & Pérez‐Juan, 2007); and treatments based on ultrasonic, pulsed electric field and high hydrostatic pressure (Corrales, Toepfl, Butz, Knorr, & Tauscher, 2008). However, many of these procedures are difficult to scale to industrial production.

Grape pomace is the main by‐product from the wine industry accounting for millions of tons each year (Food and Agriculture Organization Corporate Statistical Database FAOSTAT, 2018). Beyond its use as a source of ethanol, seed oil, and other valuable products, grape pomace is a source of phenolic substances (Kammerer, Claus, Carle, & Schieber, 2004), recognized as antioxidant and antiproliferative molecules (Lazze et al., 2009; Zhou & Raffoul, 2012), so it is highly recommended to find biomedical applications to this natural asset. Substances extracted from grapes (flavan‐3‐ols, flavonols, etc.) have demonstrated to exert numerous and different biological effects in several cancer types, both in vitro and in vivo (Gómez‐Alonso et al., 2012; Kaur, Mandair, Agarwal, & Agarwal, 2008; Kaur, Singh, Gu, Agarwal, & Agarwal, 2006; Kim et al., 2005; Li et al., 2009). Pro‐apoptotic effects and growth inhibition are among the most prominent (Agarwal et al., 2007; Inaba et al., 2008). Some studies have attributed the anticancer effects of grape extracts to epigallocatechin or procyanidin; however, the composition of these extracts is highly complex (Cavaliere et al., 2008), and therefore, other substances could be also involved. Anthocyanins, flavonols, stilbenes, and flavan‐3‐ols are among these candidates and are concentrated in the skin part of grapes, while flavan‐3‐ols belong to the only important group of polyphenols in grape seeds (Kammerer et al., 2004). In this work, we investigated the antiproliferative and cytostatic effects produced by grape pomace and grape seed extracts on colon cancer cells (Caco‐2, HT‐29) and fibroblasts (CRL2072). These effects were compared with two different extracts, crude or purified, on the same cell lines. We also tested the biological activity of the phenolic and anthocyanin subfractions of the purified extract from the red pomace to ascertain which is active. Finally, we also developed a gene expression study to determine the expression of several cell cycle regulating genes likely influenced by the extracts.

## 2 METHOD

### 2.1 Cell cultures

Colon cancer cells (Caco‐2, HT‐29) and fibroblasts (CRL2072) were used to study the biological activity of the extracts. Cells were maintained in Dulbecco's modified Eagle's Medium (DMEM) supplemented with 10% heat‐inactivated fetal bovine serum, 2 mM l‐glutamine, and penicillin (100 U/ml)/streptomycin (100 g/ml) in flasks (75 cm^2^ surface area; Sarstedt, Spain) at 37°C under 40% humidity with a 5% CO_2_ atmosphere.

### 2.2 Grape extracts

All extracts and enriched fractions were prepared from red and white grape pomaces provided by a winery located in Castilla‐La Mancha (Spain). Grape seeds were supplied by a grape pomace processing industry. Samples were stored at −20°C until used. Grape pomaces and seed were powdered while frozen by means of a grinder Stephan UMC (Stephan Food Service Equipment GMBH) in the presence of dry ice.

Crude phenolic extracts were prepared from pomaces or seeds by diluting 150 g of powdered samples in 300 ml of ethanol/water solution (50:50 v/v) at 40°C during 15 min under stirring. Solids were separated by centrifugation for 10 min at 3,500 *g* and extracted again following the same procedure. Combined supernatants were evaporated under vacuum to remove ethanol, and the aqueous concentrates were freeze‐dried to obtain powdered extracts of white grape pomace (cW), red grape pomace (cR), or grape seeds (cG).

Amberlite XAD‐7 polymeric resin was used to remove sugars, organic acids, and salts from crude extracts, thus providing purified extracts of white grape pomace (pW), red grape pomace (pR), or grape seeds (pG). A further fractioning step was carried out from pR by using two resins, MCX (mixed cation exchanger) to separate noncationic polyphenols from anthocyanins (in form of flavylium cation at acidic pHs) and C18 to eliminate in the anthocyanin fraction the acid excess used for its recovery. This procedure, adapted from Castillo‐Muñoz et al. (2010), enabled obtaining a pR subfraction enriched in anthocyanins (apR) and another one mainly containing nonanthocyanin phenolics, especially flavonols and flavan‐3‐ols (fpR). As in the case of crude extracts, all the purified fractions and subfractions were freeze‐dried to obtain powdered extracts. All the extracts were first dissolved in 1% dimethyl sulfoxide (DMSO) and then in culture media before being added to cell cultures for biological evaluation.

### 2.3 HPLC‐DAD‐ESI‐MS/MS analysis of phenolic compounds

Crude and purified extracts were analyzed by HPLC‐DAD‐ESI‐MS/MS to determine the content of anthocyanins (including 3‐glucosides of delphinidin, cyanidin, petunidin, peonidin and malvidin and their acylated derivatives), flavonols (including myricetin, quercetin, laricitrin, kaempferol, isorhamnetin, and syringetin, both as aglycones and as 3‐glycosides), hydroxycinnamic acid derivatives, flavan‐3‐ols (including monomers, dimers, and oligomers), and stilbenes (including *t*‐resveratrol, *c*‐resveratrol, *t*‐piceid, and *c*‐piceid).

HPLC separation, identification, and quantification of anthocyanins, flavonols, and hydroxycinnamic acid derivatives (Castillo‐Muñoz, Fernández‐González, Gómez‐Alonso, García‐Romero, & Hermosín‐Gutiérrez, 2009) were performed on an Agilent 1100 Series system (Agilent), equipped with DAD (G1315B) and LC/MSD Trap VL (G2445C VL) electrospray ionization mass spectrometry (ESI‐MS^n^) system, on a reversed‐phase column Zorbax Eclipse XDB‐C18 (4.6 × 250 mm; 5 μm particle; Agilent), and coupled to an Agilent Chem Station (version B.01.03) data processing station. The mass spectra data were processed with the Agilent LC/MS Trap software (version 5.3).

Flavan‐3‐ols and stilbenes were analyzed in an Agilent 1200 series system equipped with a diode array detector (DAD; Agilent) and coupled to a mass spectrometry system AB Sciex 3200 Q TRAP (Applied Biosystems) operating in Multiple Reaction Monitoring (MRM) mode; data processing was carried out with Analyst MSD software (version 1.5). Chromatographic separation was achieved on an Ascentis‐C18 column (4.6 × 150 mm; 2.7 μm particle; Supelco) and thermostated at 16°C and with a flow rate of 0.3 ml/min, according to the method described by Rebello et al. (2013).

### 2.4 Cell proliferation assay (Alamar blue)

The Alamar blue assay was used to assess the effects of extracts on Caco‐2 cell viability and proliferation. This test incorporates an oxidation–reduction indicator that changes color in response to the chemical reduction of the incubation medium resulting from cell growth. The experiments were performed following the manufacturer's instructions (Serotec). Briefly, Caco‐2 cells were seeded in 24‐well plates at 1 × 10^5^ cells/well and incubated with 500 μl DMEM containing the extracts and 10% v/v Alamar blue reagent (50 μl). Absorbances were determined at 570 and 600 nm and used to calculate cell proliferation after 2, 4, and 24 hr of incubation.

### 2.5 Cell morphology assay (Crystal violet)

The effects of extracts on cell morphology after 24 hr of incubation were studied by optical microscopy. Caco‐2, HT‐29, and fibroblasts (25 × 10^4^ cells) were seeded in 35‐mm plates with complete DMEM. After 24 hr, the medium was replaced by DMEM containing the extracts, and 24 hr later, the cells were fixed with 10% formol. After 5 min, the fixative was removed and crystal violet solution was added to the plates for 10 min. Then, it was revealed with 96% ethanol, processed to direct observation, and imaged by optical microscopy using a Nikon ECLIPSE Ti‐U microscope at 40×.

### 2.6 Cytotoxicity assay (Lactate Dehydrogenase)

In these studies, the cytotoxic effect of purified extracts and subfractions (pG, pW, pR, apR, and fpR) was evaluated by lactate dehydrogenase (LDH) release from dead and damaged cells. Caco‐2, HT‐29, and CRL2072 cells were seeded in 96‐well plates (1 × 10^4^ cells/well) containing complete DMEM, allowed to grow to semiconfluence, and incubated for 24 hr with DMEM containing different concentrations of the extracts. We used the CytoTox 96^®^ Non‐Radioactive Cytotoxicity Assay (Promega) to quantify LDH after incubation by conversion of lactate to pyruvate and the subsequent reaction thereof with a tetrazolium salt to form formazan, which was finally detected by reading absorbance at 490 nm. As a positive control, we used 10% Triton and as a negative control, DMEM without phenol red and 1% DMSO. The latter concentration of the solvent did not alter cell viability when compared with the vehicle‐free media. Two to three independent experiments were performed with four replicates for each condition (cell type and concentration).

### 2.7 Gene expression assay (qRT‐PCR)

The study of the effects of the extracts on the expression of genes involved in cell cycle was performed by real‐time quantitative PCR on Caco‐2 and HT‐29 cells incubated with the test extracts (20 μg/ml pW and pG; 5 μg/ml apR and fpR) under similar conditions as before. Pre‐designed Real‐Time ready Custom Panels (Roche) were used in which the Bax (BCL2‐associated X), Ptgs2 (prostaglandin‐endoperoxide synthase 2), Myc (myelocytomatosis viral oncogene homolog), and Cdk4 (cyclin‐dependent kinase 4) genes are represented. Gapdh (glyceraldehyde‐3‐phosphate dehydrogenase) and Rn18S1 (18S1 ribosomal RNA) were used as reference genes. The real‐time quantitative PCR was carried out in a LC480 thermocycler (Roche), and the method for calculating mRNA levels for each gene used was ΔΔCt.

### 2.8 Statistical analysis

The GraphPad PRISM software 7.0 was used for the statistical analysis. Comparisons between two experimental groups were performed with Student's *t* tests, and one‐way ANOVA or two‐way ANOVA (when applicable) followed by Dunnett's and Bonferroni post hoc tests (respectively) when the number of experimental groups was higher. Statistical significance was considered at the .05 level.

## 3 RESULTS

The phenolic composition of the extracts used in this study is presented in Table 1. The main phenolic compounds in all the extracts were flavan‐3‐ols followed by flavonols. Anthocyanins, as expected, were only present in the extracts obtained from red grape pomace and relatively important in the apR subfraction, while practically missing in the fpR subfraction. Hydroxycinnamic acid derivatives and stilbenes were found in very low quantities or not detected in the extracts. Grape seed extracts only contained flavan‐3‐ols.

**Table 1:** Phenolic composition of the different grape pomace and grape seed extracts used in this study

	ΣFlavonols (µmol/g DE^a^)	ΣAnthocyanins^b^ (µmol/g DE^a^)	ΣFlavan‐3‐ols^c^ (µmol/g DE^a^)	ΣStilbenes (µmol/g DE^a^)
cR	25.14 ± 0.27	12.26 ± 0.08	293.15 ± 10.33	0.01 ± 0.00
cW	16.60 ± 0.43	N.D.	244.51 ± 4.36	0.03 ± 0.00
cG	N.D.	N.D	752.07 ± 9.72	0.01 ± 0.00
pR	39.29 ± 3.74	19.13 ± 0.13	417.37 ± 6.30	0.04 ± 0.00
pW	46.42 ± 0.43	N.D.	808.53 ± 5.12	1.06 ± 0.03
pG	N.D.	N.D.	826.40 ± 19.43	0.08 ± 0.00
apR	27.15 ± 0.19	57.69 ± 0.41	286.37 ± 7.27	N.D.
fpR	204.05 ± 8.73	0.61 ± 0.01	599.35 ± 10.00	0.07 ± 0.00

a Results expressed as µmol of phenolic compounds per gram of dry extract as obtained after freeze dry.

b Expressed as µmol equivalents of malvidin‐3‐glucoside.

c Expressed as µmol equivalents of monomers.

**Figure 1:**
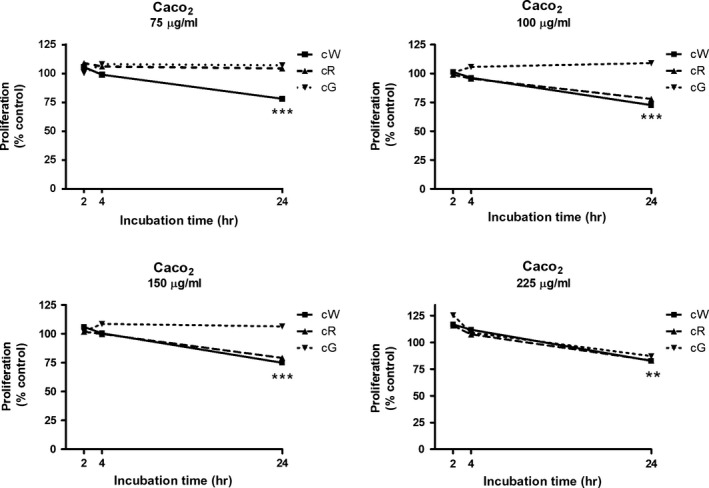
Effect of incubation with crude grape extracts (cW = crude white pomace; cR = crude red pomace; cG = crude grape seed) on Caco‐2 proliferation by Alamar Blue test. **p < .01 and ***p < .001 respect to control group

**Figure 2:**
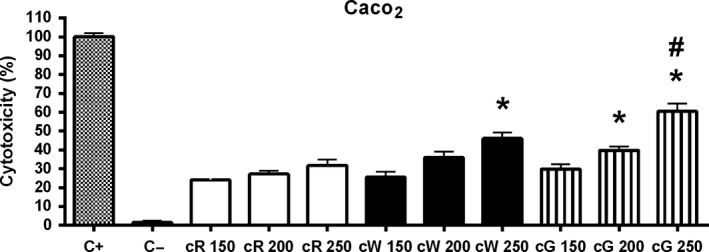
Cytotoxic effect of crude grape extracts (cR = crude red pomace; cW = crude white pomace; cG = crude grape seed) on Caco‐2 cells by the LDH test after 24‐hr incubation. C+: positive control (10% triton, maximum LDH released, *n* = 12). C−: negative control (1% DMSO in DMEM, *n* = 12). Concentrations are expressed as µg/ml. Bars represent means ± *SEM*. *p < .05 respect to its corresponding cR group. ^#^ *p* < .05 respect to its corresponding cW group

**Figure 3:**
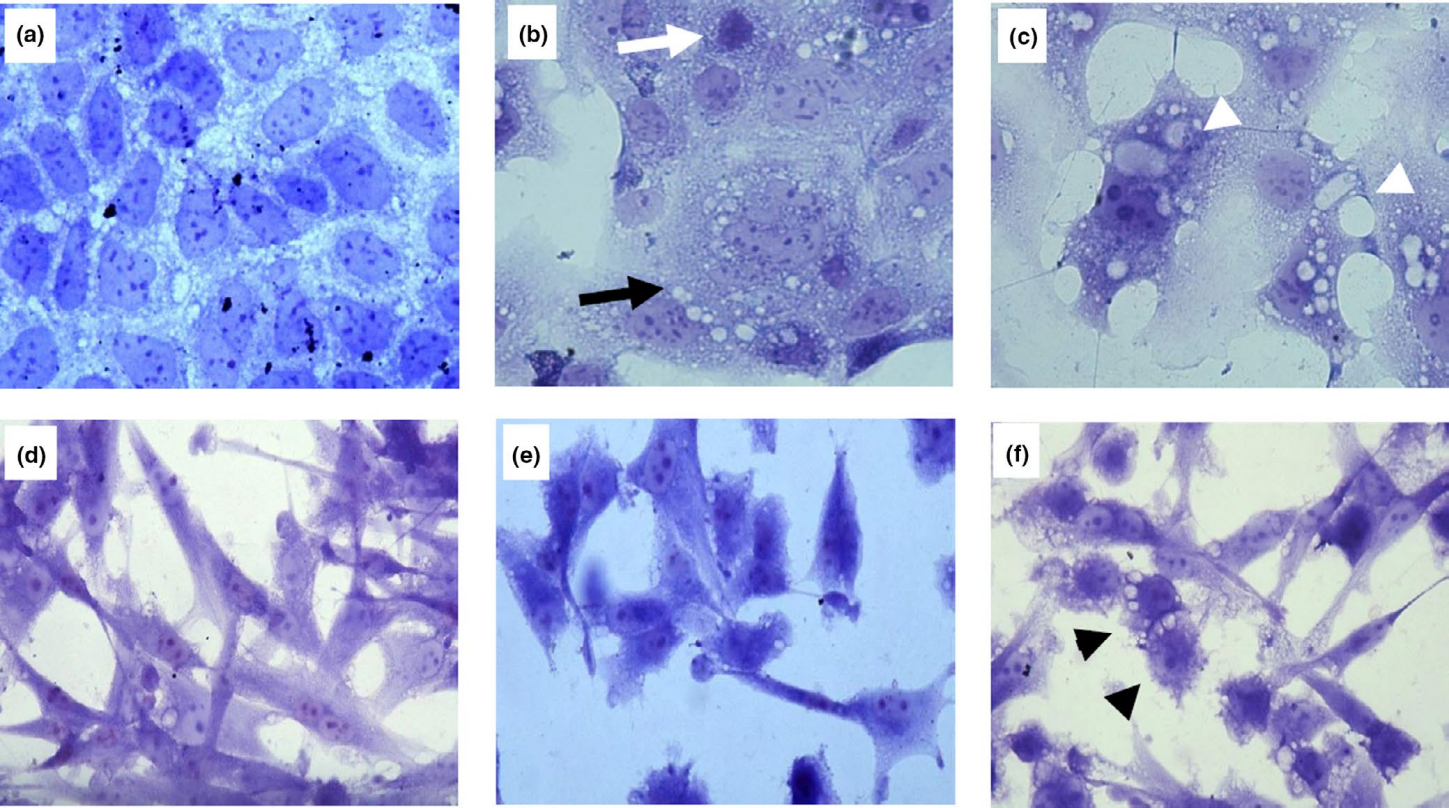
Crystal violet (40×). Cell morphology of Caco2 (a–c) and HT‐29 (d–f) cells, respectively, incubated with DMEM (a and d: control) and cG extracts (b and e: 100 μg/ml; C and F: 150 μg/ml). (b) Loss of nuclear and cytoplasmic membrane integrity (black arrow). Condensed chromatin and pycnotic nuclei (white arrow). (c) Cellular vacuolization (head white arrow). (f) Rounded cells with shortened cytoplasmic prolongations (head black arrow)

The three crude extracts examined in the Alamar blue test showed antiproliferative effects on Caco‐2 cells (Figure 1), being cW the most potent as indicated by both the maximal inhibition of proliferation (27.35%) and the minimal effective concentration (75 μg/ml). The cG extract was the least active on both parameters, and cR had an intermediate behavior. However, using the LDH method in the same cells, cG was the most cytotoxic after 24‐hr incubation, especially at the higher concentration of 250 μg/ml (Figure 2). These results correlated well with data derived from the morphological study (Figure 3, from a–c), since the incubation for 24 hr with cG elicited the appearance of swollen cells and cytoplasmic vacuolization (head white arrow), alterations that normally lead to the loss of nuclear and cytoplasmic membrane integrity (black arrow). Eventually, cells with condensed chromatin and pyknotic nuclei also appeared (white arrow). The damage produced by the extracts on HT‐29 cells (Figure 3, from d–f) was far less pronounced and similar to that observed in control fibroblasts (data not shown), where incubation with the same concentrations of cG caused the appearance of rounded cells with shortened cytoplasmic prolongations and cellular vacuolization (head black arrow) to a lesser extent than in Caco‐2.

**Figure 4:**
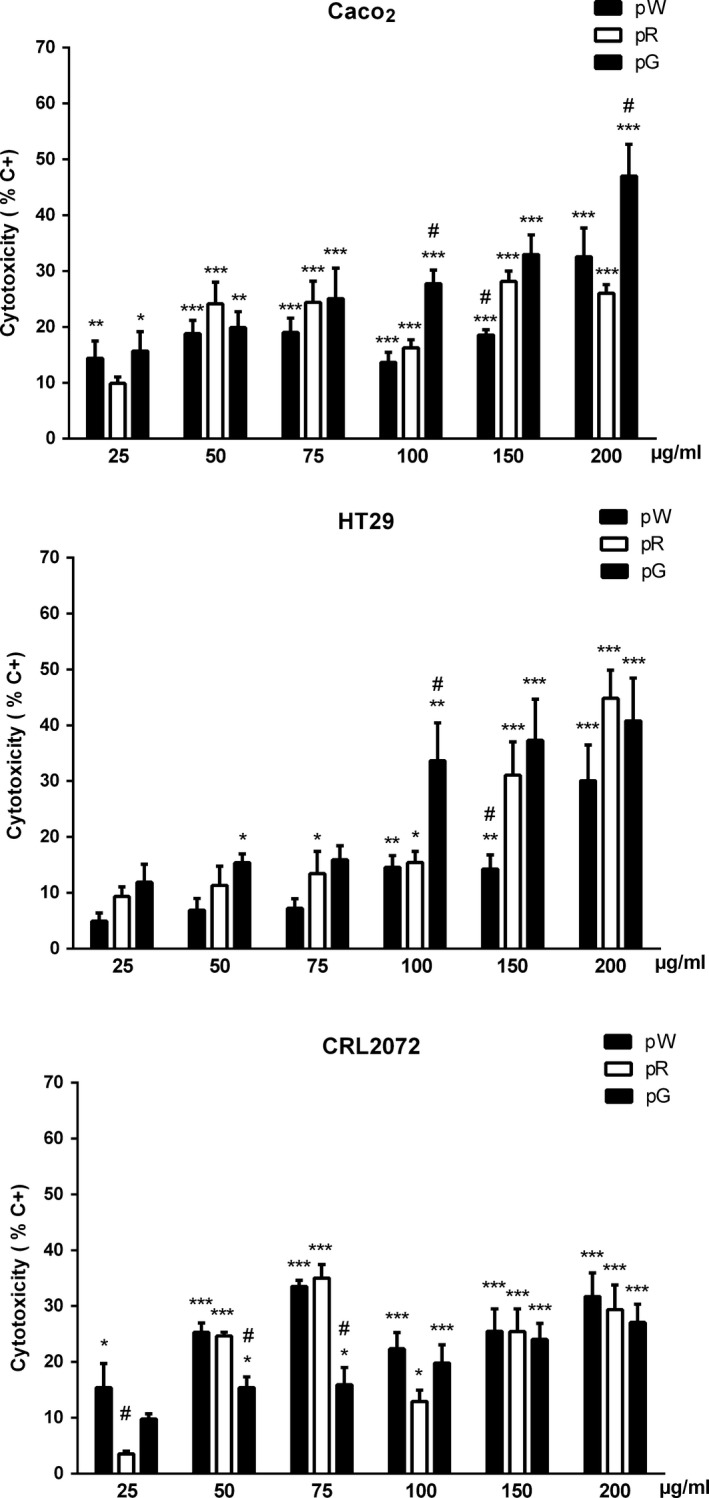
Cytotoxicity of purified extracts on Caco‐2, HT‐29, and CRL2072 cells (pW = white pomace; pR = red pomace; pG = grape seed), quantified with the LDH test. Bars represent means ± *SEM*. *p < .05, **p < .01, and ***p < .001 respect to negative control group (0.5%–1% cytotoxicity). ^#^ *p* < .05 respect to other extracts at the same concentration

**Figure 5:**
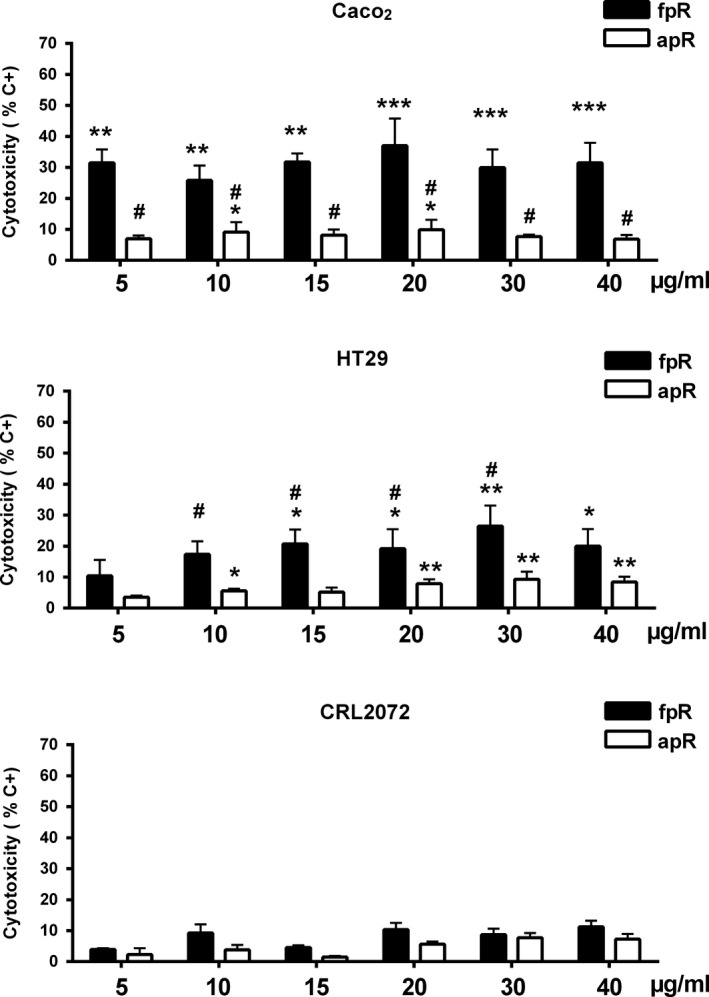
Cytotoxicity of red pomace fractions on Caco‐2, HT‐29, and CRL2072 cells (apR = anthocyanin red pomace; fpR = nonanthocyanin phenolic red pomace), quantified with the LDH test. Bars represent means ± *SEM*. *p < .05, **p < .01, and ***p < .001 respect to negative control group (0.5%–1% cytotoxicity). ^#^ *p* < .05 respect to other extracts at the same concentration

Purified grape extracts exhibited similar cytotoxicity on Caco‐2 cells, with a low concentration/effect ratio at the doses studied, with maximum effect close to 30% of maximum LDH released (positive control) in both cases. The pG extract showed a more evident concentration–response effect, approaching 50% of toxicity at the highest concentration used (Figure 4, Caco2). These results were qualitatively similar to those obtained with the crude extracts and suggest that the pG extract tends to be the most active on this cell line, as it also indicates the fact that 100 and 200 μg/ml of pG had significantly more effect than pW and pR. In the case of pR, the biological activity seems to reside almost completely in the nonanthocyanin phenolic fraction (fpR), since the anthocyanin fraction (apR) hardly had any effect. In fact, the fpR extract exhibited appreciable activity at concentrations as low as 5 μg/ml, although that effect did not increase at higher concentrations (Figure 5, Caco2). In the case of HT‐29 cells, purified extracts appeared to have a higher activity than crude extracts; pG and pR exhibited similar activity, somewhat higher than pW (Figure 4, HT‐29). Also in this case, most of the activity of pR extract seemed to reside in the nonanthocyanin phenolic fraction (Figure 5, HT‐29). The purified grape extracts affected CRL2072 viability to some extent, being their relative potencies (pW > pR > pG) different than those observed in colorectal cancer cells (Figure 4, CRL2072). The fpR fraction also showed higher specificity on colorectal cancer cells compared to fibroblasts (Figure 5, CRL2072).

**Figure 6:**
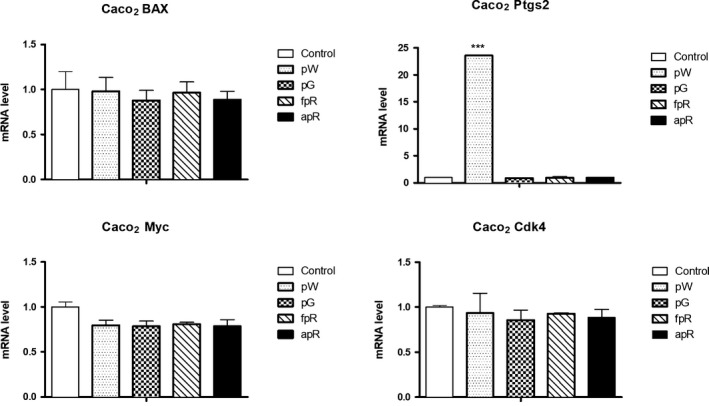
Quantification by qPCR of the effect of grape extracts (pW = white pomace; pG = grape seed; fpR = nonanthocyanin phenolic red pomace; apR = anthocyanin red pomace) on the expression of genes related to cell cycle regulation in Caco‐2 (mRNA level referred to control). Bars represent means ± *SEM*. ***p < .001 respect to control group

**Figure 7:**
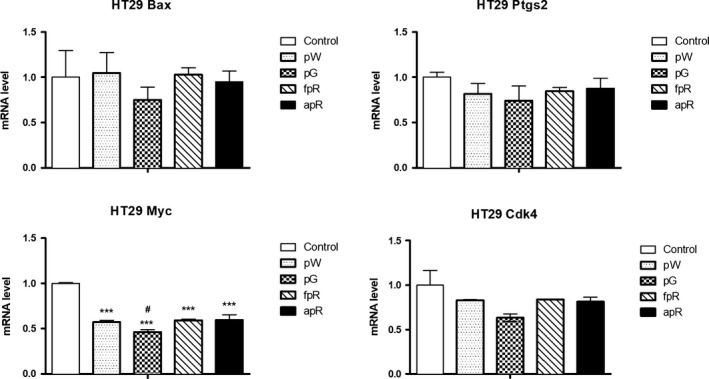
Quantification by qPCR of the effect of grape extracts (pW = white pomace; pG = grape seed; fpR = nonanthocyanin phenolic red pomace; apR = anthocyanin red pomace) on the expression of genes related to cell cycle regulation in HT‐29 (mRNA level referred to control). Bars represent means ± *SEM*. ***p < .001 respect to control group. ^#^ *p* < .05 respect to fpR and apR

Regarding the study of the cell cycle regulators, results obtained in Caco‐2 cells showed a very pronounced overexpression of Ptgs2 mRNA level after pW incubation (Figure 6). In contrast, pW and the rest of tested extracts did not affect Bax or Cdk4, and only induced a decrease on Myc mRNA expression in Caco‐2 (Figure 6). This effect was even more striking and reached statistical significance in HT‐29 cells, where all the tested extracts induced a significant decrease compared to control and pG extract proved to be the most active decreasing Myc mRNA level (Figure 7).

## 4 DISCUSSION

Purification of phenolic compounds from natural sources to homogeneity is a challenging task (Anantharaju, Gowda, Vimalambike, & Madhunapantula, 2016). Therefore, several studies have tested the ability of either crude extracts rich in phenolic compounds or the fractions containing a mixture of phenolic compounds for inhibiting cancer cell proliferation in vitro and in vivo (de Oliveira et al., 2013; Ghasemzadeh & Jaafar, 2013).

In this work, the possible cytotoxic and antiproliferative effects of crude extracts of grape pomace and seeds (cW, cR and cG) were initially evaluated in colorectal cancer cells by using the Alamar blue test. These experiments showed an antiproliferative effect on Caco‐2 cells with an order of activity cW > cR > cG. However, these effects did not correlate with the cytotoxicity of the extracts when assessed by optical microscopy, since cG appeared to be the most potent extract. These apparent discrepancies led us to expand our studies with the lactate dehydrogenase (LDH) test, which fitted much better with optical observations: Thus, cG produced the highest release of LDH, followed by cW and cR. Accordingly, we selected the LDH procedure to further progress with the study of purified extracts. Presently, we cannot provide a reason for the inconsistency between the results of morphological observations and LDH test on one hand and those of the Alamar blue test on the other. Anyhow, the results obtained do not recommend the use of the latter procedure in similar studies. If we consider that the cytotoxicity of crude extracts on the control cell line was similar, it appears that the specificity of cG on Caco‐2 cells was higher than that of cW and cR.

The results obtained with the purified extracts on Caco‐2, HT‐29, and fibroblasts cells clearly showed that the procedure used significantly enhances the biological activity of the extracts, and further supports the idea that grape seeds provide the most potent and specific effect on colorectal cell lines. In the particular case of red pomace, the biological activity of the extracts seemed to reside almost completely in the compounds that are present in nonanthocyanin phenolic fraction, since the anthocyanin fraction hardly produced any effect. Moreover, the cytotoxic activity of the nonanthocyanin phenolic fraction has a marked specificity on colorectal cancer cells, with a poor effect on fibroblasts. These data support the results obtained in other studies where phenolic compounds and flavan‐3‐ols are likely to participate in the overall grape seed extract‐mediated anticancer effect, both in vitro (Anantharaju et al., 2016; Dinicola et al., 2012; Jaganathan, Supriyanto, & Mandal, 2013; Murad, Soares Nda, Brand, Monteiro, & Teodoro, 2015; Pandey & Rizvi, 2009; Pontiki, Hadjipavlou‐Litina, Litinas, & Geromichalos, 2014; Rajendra Prasad, Karthikeyan, Karthikeyan, & Reddy, 2011) and in vivo (Zhao, Wang, Chen, & Agarwal, 1999; Zhu, Shang, Li, & Zhen, 2016). However, further digestion studies in vitro and in vivo are needed to clearly establish the bioavailability of the active compounds (D'Archivio, Filesi, Vari, Scazzocchio, & Masella, 2010).

Regarding the mRNA levels of the selected cell cycle regulator genes, we detected a high overexpression of Ptg2 after pW incubation on Caco‐2. This gene codes for cyclooxygenase‐2 (Cox2), an inducible protein that is responsible for prostaglandins biosynthesis which are involved in inflammation and mitogenesis (Jiang & Dingledine, 2013). In addition, the overexpression of Cox2 has been specifically linked to some hereditary colorectal tumors such as familial adenomatous polyposis (Burn, Mathers, & Bishop, 2013). Thus, the effect of pW on gene expression would be, at first, counterintuitive, considering the antiproliferative and cytotoxic effects observed after pW incubation in our experiments. Consequently, further experiments are needed to reveal the exact meaning of this gene regulation. In contrast, both pW and the rest of purified extracts tended to decrease Myc expression in Caco‐2. This decrease was significant in the case of HT‐29 cells, where pW proved to be effective downregulating Myc mRNA level. Myc is an oncogene whose overexpression in human tumors of several types has been described for more than two decades. It is a transcription factor that together with the MAX protein either activates or represses the expression of genes involved in cell cycle progression, angiogenesis, apoptosis, response to DNA damage, and so on (Patel, Loboda, Showe, Showe, & McMahon, 2004). The decrease in Myc expression would therefore be compatible with an antiproliferative effect of the extracts, which could also be specific on colorectal tumor cells as long as it is not seen in fibroblasts. However, further experiments are needed to reveal the exact meaning of this gene regulation.

In conclusion, our results suggest a likely antitumor effect of the extracts studied, where grape seed extracts and the nonanthocyanin phenolic fraction tended to be the most active on colorectal cancer cell lines. However, purification and fractionation should be continued to discover the most active molecule.

## CONFLICT OF INTEREST

The authors declare that they do not have any conflict of interest.

## ETHICAL APPROVAL

Ethical Review: This study does not involve any human or animal testing.

## References

[fsn31150-bib-0001] Agarwal, C. , Veluri, R. , Kaur, M. , Chou, S. C. , Thompson, J. A. , & Agarwal, R. (2007). Fractionation of high molecular weight tannins in grape seed extract and identification of procyanidin B2–3,3'‐di‐O‐gallate as a major active constituent causing growth inhibition and apoptotic death of DU145 human prostate carcinoma cells. Carcinogenesis, 28(7), 1478–1484. 10.1093/carcin/bgm045 17331955

[fsn31150-bib-0002] Amico, V. , Barresi, V. , Chillemi, R. , Condorelli, D. F. , Sciuto, S. , Spatafora, C. , & Tringali, C. (2009). Bioassay‐guided isolation of antiproliferative compounds from grape (*Vitis vinifera*) stems. Natural Products Communications, 4(1), 27–34. 19370870

[fsn31150-bib-0003] Anantharaju, P. G. , Gowda, P. C. , Vimalambike, M. G. , & Madhunapantula, S. V. (2016). An overview on the role of dietary phenolics for the treatment of cancers. Nutrition Journal, 15(1), 99 10.1186/s12937-016-0217-2 27903278 PMC5131407

[fsn31150-bib-0004] Burn, J. , Mathers, J. , & Bishop, D. T. (2013). Genetics, inheritance and strategies for prevention in populations at high risk of colorectal cancer (CRC). Recent Results in Cancer Research, 191, 157–183. 10.1007/978-3-642-30331-9_9 22893205

[fsn31150-bib-0005] Campos, L. M. , Leimann, F. V. , Pedrosa, R. C. , & Ferreira, S. R. (2008). Free radical scavenging of grape pomace extracts from Cabernet sauvingnon (*Vitis vinifera*). Bioresource Technology, 99, 8413–8420. 10.1016/j.biortech.2008.02.058 18445523

[fsn31150-bib-0006] Castillo‐Muñoz, N. , Fernández‐González, M. , Gómez‐Alonso, S. , García‐Romero, E. , & Hermosín‐Gutiérrez, I. (2009). Red‐color related phenolic composition of garnacha tintorera (*Vitis vinifera* L.) grapes and red wines. Journal of Agriculture and Food Chemistry, 57, 7883–7891. 10.1021/jf9002736 19673489

[fsn31150-bib-0007] Castillo‐Muñoz, N. , Winterhalter, P. , Weber, F. , Gómez, M. V. , Gómez‐Alonso, S. , García‐Romero, E. , & Hermosín‐Gutiérrez, I. (2010). Structure elucidation of peonidin 3,7‐O‐beta‐diglucoside, a chemical marker of garnacha tintorera (*V. vinifera* L.) grapes and wines. Journal of Agriculture and Food Chemistry, 58, 11105–11111. 10.1021/jf102578u 20866031

[fsn31150-bib-0008] Cavaliere, C. , Foglia, P. , Gubbiotti, R. , Sacchetti, P. , Samperi, R. , & Lagana, A. (2008). Rapid‐resolution liquid chromatography/mass spectrometry for determination and quantitation of polyphenols in grape berries. Rapid Communications in Mass Spectrometry, 22(20), 3089–3099. 10.1002/rcm.3705 18819110

[fsn31150-bib-0009] Corrales, M. , Toepfl, S. , Butz, P. , Knorr, D. , & Tauscher, B. (2008). Extraction of anthocyanins from grape by‐products assisted by ultrasonics, high hydrostatic pressure or pulsed electric fields: A comparison. Innovative Food Science & Emerging Technologies, 9, 85–91. 10.1016/j.ifset.2007.06.002

[fsn31150-bib-0010] Cruz, J. M. , Conde, E. , Domínguez, H. , & Parajó, J. C. (2007). Thermal stability of antioxidants obtained from wood and industrial wastes. Food Chemistry, 100, 1059–1064. 10.1016/j.foodchem.2005.11.012

[fsn31150-bib-0011] Cruz, J. M. , Domínguez, H. , & Parajó, J. C. (2004). Assessment of the production of antioxidants from winemaking waste solids. Journal of Agricultural and Food Chemistry, 52(18), 5612–5620. 10.1021/jf049376c 15373401

[fsn31150-bib-0012] D'Archivio, M. , Filesi, C. , Vari, R. , Scazzocchio, B. , & Masella, R. (2010). Bioavailability of the polyphenols: Status and controversies. International Journal of Molecular Sciences, 11(4), 1321–1342. 10.3390/ijms11041321 20480022 PMC2871118

[fsn31150-bib-0013] de Oliveira, C. , Comunello, L. , Maciel, É. , Giubel, S. , Bruno, A. , Chiela, E. , … Gosmann, G. (2013). The inhibitory effects of phenolic and terpenoid compounds from *Baccharis trimera* in Siha cells: Differences in their activity and mechanism of action. Molecules, 18(9), 11022–11032. 10.3390/molecules180911022 24022763 PMC6270023

[fsn31150-bib-0014] Dinicola, S. , Cucina, A. , Pasqualato, A. , D'Anselmi, F. , Proietti, S. , Lisi, E. , … Bizzarri, M. (2012). Antiproliferative and apoptotic effects triggered by Grape Seed Extract (GSE) versus epigallocatechin and procyanidins on colon cancer cell lines. International Journal of Molecular Sciences, 13(1), 651–664. 10.3390/ijms13010651 22312277 PMC3269711

[fsn31150-bib-0015] Faria, A. , Calhau, C. , de Freitas, V. , & Mateus, N. (2006). Procyanidins as antioxidants and tumor cell growth modulators. Journal of Agriculture and Food Chemistry, 54(6), 2392–2397. 10.1021/jf0526487 16536624

[fsn31150-bib-0016] Fresco, P. , Borges, F. , Marques, M. P. , & Diniz, C. (2010). The anticancer properties of dietary polyphenols and its relation with apoptosis. Current Pharmaceutical Design, 16(1), 114–134. 20214622 10.2174/138161210789941856

[fsn31150-bib-0047] Food and Agriculture Organization Corporate Statistical Database (FAOSTAT) (2018). https://www.fao.org/statistics/en/ [last accessed 2 February 2019].

[fsn31150-bib-0017] Ghasemzadeh, A. , & Jaafar, H. Z. (2013). Profiling of phenolic compounds and their antioxidant and anticancer activities in pandan (*Pandanus amaryllifolius* Roxb.) extracts from different locations of Malaysia. BMC Complementary and Alternative Medicine, 13, 341 10.1186/1472-6882-13-341 24289290 PMC4220543

[fsn31150-bib-0018] Glade, M. J. (1999). Food, nutrition, and the prevention of cancer: a global perspective. American Institute for Cancer Research/World Cancer Research Fund, American Institute for Cancer Research, 1997. Nutrition, 15(6), 523–526. 10378216 10.1016/s0899-9007(99)00021-0

[fsn31150-bib-0019] Gómez‐Alonso, S. , Collins, V. J. , Vauzour, D. , Rodríguez‐Mateos, A. , Corona, G. , & Spencer, J. P. E. (2012). Inhibition of colon adenocarcinoma cell proliferation by flavonols is linked to a G2/M cell cycle block and reduction in cyclin D1 expression. Food Chemistry, 130, 493–500. 10.1016/j.foodchem.2011.07.033

[fsn31150-bib-0020] Gonzalez‐Paramas, A. M. , Esteban‐Ruano, S. , Santos‐Buelga, C. , de Pascual‐Teresa, S. , & Rivas‐Gonzalo, J. C. (2004). Flavanol content and antioxidant activity in winery byproducts. Journal of Agriculture and Food Chemistry, 52(2), 234–238. 10.1021/jf0348727 14733501

[fsn31150-bib-0021] Inaba, H. , Nagaoka, Y. , Kushima, Y. , Kumagai, A. , Matsumoto, Y. , Sakaguchi, M. , … Uesato, S. (2008). Comparative examination of anti‐proliferative activities of (‐)‐epigallocatechin gallate and (–)‐epigallocatechin against HCT116 colorectal carcinoma cells. Biological and Pharmaceutical Bulletin, 31(1), 79–84. 10.1248/bpb.31.79 18175946

[fsn31150-bib-0022] Jaganathan, S. K. , Supriyanto, E. , & Mandal, M. (2013). Events associated with apoptotic effect of p‐Coumaric acid in HCT‐15 colon cancer cells. World Journal of Gastroenterology, 19(43), 7726–7734. 10.3748/wjg.v19.i43.7726 24282361 PMC3837272

[fsn31150-bib-0023] Jiang, J. , & Dingledine, R. (2013). Role of prostaglandin receptor EP2 in the regulations of cancer cell proliferation, invasion, and inflammation. Journal of Pharmacology and Experimental Therapeutics, 344(2), 360–367. 10.1124/jpet.112.200444 23192657 PMC3558819

[fsn31150-bib-0024] Kammerer, D. , Claus, A. , Carle, R. , & Schieber, A. (2004). Polyphenol screening of pomace from red and white grape varieties (*Vitis vinifera* L.) by HPLC‐DAD‐MS/MS. Journal of Agriculture and Food Chemistry, 52(14), 4360–4367. 10.1021/jf049613b 15237937

[fsn31150-bib-0025] Kaur, M. , Mandair, R. , Agarwal, R. , & Agarwal, C. (2008). Grape seed extract induces cell cycle arrest and apoptosis in human colon carcinoma cells. Nutrition and Cancer, 60(Suppl 1), 2–11. 10.1080/01635580802381295 19003575 PMC2597484

[fsn31150-bib-0026] Kaur, M. , Singh, R. P. , Gu, M. , Agarwal, R. , & Agarwal, C. (2006). Grape seed extract inhibits in vitro and in vivo growth of human colorectal carcinoma cells. Clinical Cancer Research, 12(20 Pt 1), 6194–6202. 10.1158/1078-0432.CCR-06-1465 17062697

[fsn31150-bib-0027] Khan, N. , Adhami, V. M. , & Mukhtar, H. (2009). Review: Green tea polyphenols in chemoprevention of prostate cancer: Preclinical and clinical studies. Nutrition and Cancer, 61(6), 836–841. 10.1080/01635580903285056 20155624 PMC2991093

[fsn31150-bib-0028] Kim, Y. J. , Park, H. J. , Yoon, S. H. , Kim, M. J. , Leem, K. H. , Chung, J. H. , & Kim, H. K. (2005). Anticancer effects of oligomeric proanthocyanidins on human colorectal cancer cell line, SNU‐C4. World Journal of Gastroenterology, 11(30), 4674–4678. 10.3748/wjg.v11.i30.4674 16094708 PMC4615409

[fsn31150-bib-0029] Lamoral‐Theys, D. , Pottier, L. , Dufrasne, F. , Neve, J. , Dubois, J. , Kornienko, A. , … Ingrassia, L. (2010). Natural polyphenols that display anticancer properties through inhibition of kinase activity. Current Medicinal Chemistry, 17(9), 812–825. 20156174 10.2174/092986710790712183

[fsn31150-bib-0030] Lazze, M. C. , Pizzala, R. , Gutierrez Pecharroman, F. J. , Gaton Garnica, P. , Antolin Rodriguez, J. M. , Fabris, N. , & Bianchi, L. (2009). Grape waste extract obtained by supercritical fluid extraction contains bioactive antioxidant molecules and induces antiproliferative effects in human colon adenocarcinoma cells. Journal of Medicinal Food, 12(3), 561–568. 10.1089/jmf.2008.0150 19627204

[fsn31150-bib-0031] Li, L. , Adams, L. S. , Chen, S. , Killian, C. , Ahmed, A. , & Seeram, N. P. (2009). Eugenia jambolana Lam. berry extract inhibits growth and induces apoptosis of human breast cancer but not non‐tumorigenic breast cells. Journal of Agriculture and Food Chemistry, 57(3), 826–831. 10.1021/jf803407q PMC2680249 19166352

[fsn31150-bib-0032] Liu, H. L. , Jiang, W. B. , & Xie, M. X. (2010). Flavonoids: Recent advances as anticancer drugs. Recent Patents on Anti‐Cancer Drug Discovery, 5(2), 152–164. 20088766 10.2174/157489210790936261

[fsn31150-bib-0033] Luque‐Rodríguez, J. M. , Luque de Castro, M. D. , & Pérez‐Juan, P. (2007). Dynamic superheated liquid extraction of anthocyanins and other phenolics from red grape skins of winemaking residues. Bioresource Technology, 98, 2705–2713. 10.1016/j.biortech.2006.09.019 17092712

[fsn31150-bib-0034] Maier, T. , Göppert, A. , Kammerer, D. R. , Schieber, A. , & Carle, R. (2008). Optimization of a process for enzyme‐assisted pigment extraction from grape (*Vitis vinifera* L.) pomace. European Food Research and Technology, 227, 267–275. 10.1007/s00217-007-0720-y

[fsn31150-bib-0035] Meyer, A. S. , Jepsen, S. M. , & Sørensen, N. S. (1998). Enzymatic release of antioxidants for human low‐density lipoprotein from grape pomace. Journal of Agricultural and Food Chemistry, 46(7), 2439–2446. 10.1021/jf971012f

[fsn31150-bib-0036] Murad, L. D. , Soares Nda, C. , Brand, C. , Monteiro, M. C. , & Teodoro, A. J. (2015). Effects of caffeic and 5‐caffeoylquinic acids on cell viability and cellular uptake in human colon adenocarcinoma cells. Nutrition and Cancer, 67(3), 532–542. 10.1080/01635581.2015.1004736 25803129

[fsn31150-bib-0037] Pandey, K. B. , & Rizvi, S. I. (2009). Plant polyphenols as dietary antioxidants in human health and disease. Oxidative Medicine and Cellular Longevity, 2(5), 270–278. 10.4161/oxim.2.5.9498 20716914 PMC2835915

[fsn31150-bib-0038] Patel, J. H. , Loboda, A. P. , Showe, M. K. , Showe, L. C. , & McMahon, S. B. (2004). Analysis of genomic targets reveals complex functions of MYC. Nature Reviews Cancer, 4(7), 562–568. 10.1038/nrc1393 15229481

[fsn31150-bib-0039] Pinelo, M. , Ruiz‐Rodríguez, A. , Sineiro, J. , Señoráns, F. J. , Reglero, G. , & Nuñez, M. J. (2007). Supercritical fluid and solid–liquid extraction of phenolic antioxidants from grape pomace: A comparative study. European Food Research and Technology, 226, 199–205. 10.1007/s00217-006-0526-3

[fsn31150-bib-0040] Pontiki, E. , Hadjipavlou‐Litina, D. , Litinas, K. , & Geromichalos, G. (2014). Novel cinnamic acid derivatives as antioxidant and anticancer agents: Design, synthesis and modeling studies. Molecules, 19(7), 9655–9674. 10.3390/molecules19079655 25004073 PMC6270778

[fsn31150-bib-0041] Rajendra Prasad, N. , Karthikeyan, A. , Karthikeyan, S. , & Reddy, B. V. (2011). Inhibitory effect of caffeic acid on cancer cell proliferation by oxidative mechanism in human HT‐1080 fibrosarcoma cell line. Molecular and Cellular Biochemistry, 349(1–2), 11–19. 10.1007/s11010-010-0655-7 21116690

[fsn31150-bib-0042] Rebello, L. P. G. , Lago‐Vanzela, E. S. , Barcia, M. T. , Ramos, A. M. , Stringheta, P. C. , Da‐Silva, R. , … Hermosín‐Gutiérrez, I. (2013). Phenolic composition of the berry parts of hybrid grape cultivar BRS Violeta (BRS Rubea×IAC 1398–21) using HPLC–DAD–ESI‐MS/MS. Food Research International, 54, 354–366. 10.1016/j.foodres.2013.07.024

[fsn31150-bib-0043] Sun, C. L. , Yuan, J. M. , Koh, W. P. , & Yu, M. C. (2006). Green tea, black tea and breast cancer risk: A meta‐analysis of epidemiological studies. Carcinogenesis, 27(7), 1310–1315. 10.1093/carcin/bgi276 16311246

[fsn31150-bib-0044] Zhao, J. , Wang, J. , Chen, Y. , & Agarwal, R. (1999). Anti‐tumor‐promoting activity of a polyphenolic fraction isolated from grape seeds in the mouse skin two‐stage initiation‐promotion protocol and identification of procyanidin B5–3'‐gallate as the most effective antioxidant constituent. Carcinogenesis, 20(9), 1737–1745. 10.1093/carcin/20.9.1737 10469619

[fsn31150-bib-0045] Zhou, K. , & Raffoul, J. J. (2012). Potential anticancer properties of grape antioxidants. Journal of Oncology, 2012, 803294 10.1155/2012/803294 22919383 PMC3420094

[fsn31150-bib-0046] Zhu, B. , Shang, B. , Li, Y. , & Zhen, Y. (2016). Inhibition of histone deacetylases by trans‐cinnamic acid and its antitumor effect against colon cancer xenografts in athymic mice. Molecular Medicine Reports, 13(5), 4159–4166. 10.3892/mmr.2016.5041 27035417 PMC4838168

